# Multidrug Resistance-Associated Protein 2 (MRP2) Mediated Transport of Oxaliplatin-Derived Platinum in Membrane Vesicles

**DOI:** 10.1371/journal.pone.0130727

**Published:** 2015-07-01

**Authors:** Khine Myint, Yan Li, James Paxton, Mark McKeage

**Affiliations:** 1 Department of Pharmacology and Clinical Pharmacology, University of Auckland, Auckland, New Zealand; 2 Auckland Cancer Society Research Centre, University of Auckland, Auckland, New Zealand; 3 School of Applied Sciences, Auckland University of Technology, Auckland, New Zealand; University of Technology Sydney, AUSTRALIA

## Abstract

The platinum-based anticancer drug oxaliplatin is important clinically in cancer treatment. However, the role of multidrug resistance-associated protein 2 (MRP2) in controlling oxaliplatin membrane transport, *in vivo* handling, toxicity and therapeutic responses is unclear. In the current study, preparations of MRP2-expressing and control membrane vesicles, containing inside-out orientated vesicles, were used to directly characterise the membrane transport of oxaliplatin-derived platinum measured by inductively coupled plasma mass spectrometry. Oxaliplatin inhibited the ATP-dependent accumulation of the model MRP2 fluorescent probe, 5(6)-carboxy-2,'7'-dichlorofluorescein, in MRP2-expressing membrane vesicles. MRP2-expressing membrane vesicles accumulated up to 19-fold more platinum during their incubation with oxaliplatin and ATP as compared to control membrane vesicles and in the absence of ATP. The rate of ATP-dependent MRP2-mediated active transport of oxaliplatin-derived platinum increased non-linearly with increasing oxaliplatin exposure concentration, approaching a plateau value (Vmax) of 2680 pmol Pt/mg protein/10 minutes (95%CI, 2010 to 3360 pmol Pt/mg protein/10 minutes), with the half-maximal platinum accumulation rate (Km) at an oxaliplatin exposure concentration of 301 μM (95% CI, 163 to 438 μM), in accordance with Michaelis-Menten kinetics (r^2^ = 0.954). MRP2 inhibitors (myricetin and MK571) reduced the ATP-dependent accumulation of oxaliplatin-derived platinum in MRP2-expressing membrane vesicles in a concentration-dependent manner. To identify whether oxaliplatin, or perhaps a degradation product, was the likely substrate for this active transport, HPLC studies were undertaken showing that oxaliplatin degraded slowly in membrane vesicle incubation buffer containing chloride ions and glutathione, with approximately 95% remaining intact after a 10 minute incubation time and a degradation half-life of 2.24 hours (95%CI, 2.08 to 2.43 hours). In conclusion, MRP2 mediates the ATP-dependent active membrane transport of oxaliplatin-derived platinum. Intact oxaliplatin and its anionic monochloro oxalate ring-opened intermediate appear likely candidates as substrates for MRP2-mediated transport.

## Introduction

The platinum-based anticancer drug oxaliplatin, and its combination therapies, are clinically important for treating colorectal cancer and other gastrointestinal malignancies [1]. However, oxaliplatin-based chemotherapy is limited by poor efficacy and high toxicity in a proportion of treated patients, who exhibit disease progression or severe adverse drug reactions early after the commencement of therapy [2–5]. The pharmacological basis of these variable clinical responses to oxaliplatin is currently unclear. Prior to inducing cytotoxicity in tumour or normal cells, oxaliplatin must transit through cell membranes before accessing and reacting with DNA, forming DNA-platinum adducts and inducing cell death and cell cycle arrest [6]. As oxaliplatin is highly hydrophilic [7, 8] and chemically transforms into charged intermediates in biological solutions [9], its inherent capacity for crossing cell membranes by passive diffusion may be limited. Recent evidence has pointed to alternative membrane transport mechanisms involving transporter proteins whereby oxaliplatin moves into and out of cells [10–21]. As this field of research is relatively new, it seems likely that many interactions involving oxaliplatin and membrane transporter proteins remain to be characterised.

The role of multidrug resistance-associated protein 2 (MRP2) in the membrane transport of oxaliplatin-derived platinum is currently unclear. MRP2 is an integral 190 kDa protein, encoded for by the *ABCC2* gene and also known as canalicular multispecific organic anion transport (cMOAT) [22]. The MRP2 protein consists of two ATP-binding domains and 17 transmembrane regions in its amino acid sequence, and functions in the transport of substrates across cell membranes using energy derived from ATP hydrolysis [22]. The MRP2 protein is expressed at major physiological barriers, including the biliary canalicular membranes of hepatocytes and apical membranes of renal proximal tubular cells, where it functions in the excretion of a wide range of structurally diverse endogenous and exogenous small molecular weight compounds into the bile and urine, respectively [22]. MRP2 is also known for being expressed by tumour cells and tissues, and contributing to multidrug resistance [22]. Its functional genetic variations contribute to altered drug handling [23]. Early work suggested that MRP2 may be an efflux transporter of cisplatin [24, 25]. Further evidence for interactions between MRP2 and cisplatin subsequently came from studies of recombinant cell lines, preclinical cell lines and *in vivo* tumour models and clinical-association studies, as reviewed in Liu et al. [11]. However, as far as we are aware, there have been no studies to date directly addressing whether oxaliplatin or platinum derived from oxaliplatin is transported by MRP2. Recent reports of positive clinical-association studies linking *ABCC2* genotype and MRP2 expression level with patient responses to oxaliplatin [26, 27], and of MRP2 determining oxaliplatin antitumor responses and resistance in preclinical models [28–30], have added further to the urgency for fundamental understanding of this transport mechanism.

These considerations led us to undertake the present study to determine if MRP2 could transport platinum derived from oxaliplatin *in vitro*. Human MRP2 protein expressing inside-out orientated membrane vesicles, prepared from Sf9 insect cells transiently transfected with the *ABCC2* gene, were used for these studies. Such membrane vesicle preparations have advantages for studies of drug efflux transporter mechanisms [31], such as experimental control over free drug concentration at the cytoplasmic transporter protein substrate binding sites, in a way that is not possible in whole cells. Inductively coupled plasma mass spectrometry (ICPMS) was used to measure membrane vesicle accumulation of platinum in this study. ICPMS is highly sensitive and specific for detecting platinum in biological matrices [32, 33] but does not distinguish intact oxaliplatin from other forms of platinum derived from oxaliplatin that may become associated with membrane vesicles during their incubation with oxaliplatin. As oxaliplatin degrades in aqueous solutions containing chloride ions [9, 34] or glutathione [35, 36], it could not be assumed that oxaliplatin had remained intact in the membrane vesicle incubation buffer or was the form of platinum transported by MRP2 even though short incubation times of between 5 to 20 minutes were used. Therefore, the stability and degradation of oxaliplatin in membrane vesicle incubation buffer was studied using a validated high-performance liquid chromatography-ultraviolet detection (HPLC-UV) method [34]. A non-sulphur compound (myricetin) was used as a MRP2 inhibitor [37–39] for these studies to avoid potential confounding effects of platinum binding to sulphur residues.

## Materials and Methods

### Chemicals

Oxaliplatin powder (Actavis New Zealand) was dissolved in 5% glucose solution, sonicated and filtered with a 0.22 μm Millipore filtration system (Bedford, USA) and stored at -20°C. The membrane vesicle incubation buffer solution consisted of 50 mM MOPS-Tris, 70 mM KCl, 7.5 mM MgCl_2_ and was freshly prepared using MOPS-Tris, KCl and MgCl_2_ powders dissolved in Milli-Q grade water (Millipore, Bedford, USA). All other chemicals were sourced from Sigma-Aldrich, St Louis, MO, USA. Stock solutions of 5(6)-carboxy-2,'7'-dichlorofluorescein (CDCF), myricetin and MK571 were prepared at concentrations 1000 times higher than working solutions in dimethylformamide and stored at -20°C.

### Membrane vesicle transport assays

MRP2-expressing and control inside-out membrane vesicles (5 mg total protein/ml) prepared from Sf9 (Spodoptera frugiperda) insect cells, transiently transfected with the human *ABCC2* gene encoding MRP2 protein or an empty vector control, respectively, along with membrane vesicle incubation buffer solution and stopping buffer solution, were obtained from GenoMembrane, Co., Ltd (Yokohama, Japan). Vesicles were stored at -80°C until use. Membrane vesicular transport assays were performed according to the manufacturer’s protocol with final concentrations shown below in brackets and some modifications. Briefly, MRP2-expressing and control vesicles (40 μg protein/8 μL) were incubated with 11.6 μL of membrane vesicle incubation buffer in an Eppendorf tube at 37°C for 5 min. Then, a pre-warmed reaction mixture containing the test substrate, ATP or AMP (4mM) and glutathione (2 mM) was added to a final volume of 40 μL. After the designated incubation time, the reaction was stopped by adding 200 μL of ice-cold stopping buffer solution containing 40 mM MOPS-Tris and 70 mM KCl and mixing. The reaction mixture was then placed into pre-wet, 96-well glass-fibre filter plates (MultiScreenHTS-FB plate, Merck-Millipore, MA, USA) fitted onto a suction filtration device (MultiScreenHTS Vacuum Manifold, Merck-Millipore, MA, USA) connected to a vacuum pump. The wells were filtered and washed twice with ice-cold stopping buffer while under vacuum. The bottom of the plate was then thoroughly wiped, followed by the addition of 10% sodium dodecyl sulphate (SDS) (100 μL) to each well for 30 min to lyse the membrane vesicles trapped on the filter plate and elute the encapsulated test substrate. The filter plate was then centrifuged (1000 rpm for 1 min) twice to collect the dissolved membrane vesicles and eluted substrate into an underlying 96-well plate.

To validate the MRP2-mediated membrane transport activity, MRP2-expressing and control membrane vesicles were exposed to a model fluorescent probe, CDCF (5 μM) for 5 min in the presence or absence of ATP. After incubation and filtration, the membrane vesicle CDCF accumulation was measured as follows. Dissolved membrane vesicles were collected in a black fluorometer plate and treated with 0.1N NaOH (100 μl) for 5 min. The fluorescent intensity of the solution was then measured at 495 nm excitation and 529 nm emission wavelengths with filters of 485 and 535 nm wavelengths, respectively, using a microplate multi-mode plate reader (Synergy HT, BioTek Instruments, Inc., USA). CDCF solution alone (4 μL 50 μM CDCF dissolved in 36 μL membrane vesicle incubation buffer) was also added to the wells of the filter plate, treated with 100 μL 10% SDS solution for 30 min, centrifuged and treated with 100 μL 0.1N NaOH solution to give the total fluorescent intensity of 200 pmol CDCF. A background fluorescent reading was obtained by measuring the fluorescence intensity of the wells containing 100 μL 10% SDS solution and 100 μL 0.1N NaOH. All the fluorescent readings were corrected by subtracting this background reading. The amount of CDCF in samples (pmol) was calculated from the ratio of the fluorescence intensity of the sample divided by the fluorescence intensity of the 200 pmol standard, multiplied by 200 pmol.

The membrane vesicle accumulation of platinum was measured as follows. After collection in a blank plate, the dissolved membrane vesicles were digested with 70% nitric acid (2:1 volume ratio of nitric acid to membrane vesicle solution) at room temperature overnight followed by heating at 95°C for 2 h. After determination of protein content using a modified tyrosine nitration assay [40], the membrane digests were diluted in MilliQ water containing thallium 50 pbb as an internal standard. The platinum content of the digested vesicles was then measured using a Varian 820MS ICPMS from Agilent Technologies Inc., Santa Clara, CA, USA) at LabPLUS (Auckland, New Zealand). Platinum counts were corrected by subtracting background counts from blank membrane vesicle samples and dividing the resulting value by the thallium internal standard counts. The platinum concentration of each sample was calculated from the platinum to thallium count ratios using the standard curve method. The calibration curve was generated with known concentrations of platinum stock solutions dissolved in the same matrix as the membrane samples and was included in each ICP-MS run along with the test samples. Quality controls (QCs) from the lower, middle and upper concentrations of the standard curve range were independently generated and included in each assay run to calculate the accuracy and precision and determine the reliability of each run. The platinum measurements were regarded as acceptable if the standard calibration curve was linear, the QCs and at least 3 concentration values of the standard curve were within ±15% precision and accuracy with a lower limit of quantification (LLOQ) value within ± 20% of precision and accuracy. The limit of detection (LOD) and LLOQ were found to be 0.3 ppb and 1.0 ppb of platinum, respectively. The ATP-dependent component of membrane vesicle platinum accumulation was derived by subtracting the amount of platinum accumulating in the absence of ATP from that in the presence of ATP at each respective oxaliplatin exposure time and concentration.

### HPLC studies

Oxaliplatin, Pt(DACH)Cl_2_, glutathione and degradation products in the membrane vesicle incubation buffer were detected using a previously validated HPLC-UV method [34]. A 50 μl incubation sample was injected onto a μBondapak C18 3.9 x 300 mm column (from Waters, Massachusetts, USA) using a 0.5 ml/ min mobile phase of 6% methanol in Milli-Q water adjusted to pH 2.5 with 10% triflic acid (v/v). The HPLC was a Hewlett Packard HP1200 online system with an HP1200 binary pump and degasser, an autosampler from Wilmington, DE, USA, and a Lambda-max model 480 LC UV detector from Millipore Waters (Lane Cove, Australia). The eluting compounds of interest were detected by absorbance at 210 nm (bandwidth of 10 nm) referenced to 550 nm (bandwidth of 50 nm). The column was washed with 60:40 methanol/water for at least 30 min followed by Milli-Q water for at least 30 min, before and after each run. Prior to each run, the column was equilibrated with the mobile phase for at least 30 min. For stability studies, 100 μM oxaliplatin was incubated in the membrane vesicle incubation buffer (pH 7.4, 37°C) with or without 2 mM glutathione. Samples were collected at designated incubation times up to 24 h and immediately injected onto the HPLC column. Oxaliplatin concentrations were determined by comparison of their peak areas with that of an authentic standard. Other compounds were detected semi-quantitatively based on the retention times and peak areas of authentic standards.

### Data analysis

Data were assessed visually and analysed by descriptive statistics. The statistical significance of differences between groups was analysed by one-way or two-way ANOVA tests followed by Tukey’s multiple comparison post-tests. *P*-values of < 0.05 indicated statistical significance. Trends in kinetic data were analysed by nonlinear regression fits. Statistical analyses were performed using Prism 6 software (GraphPad, San Diego, CA, USA).

## Results

**Fig 1:**
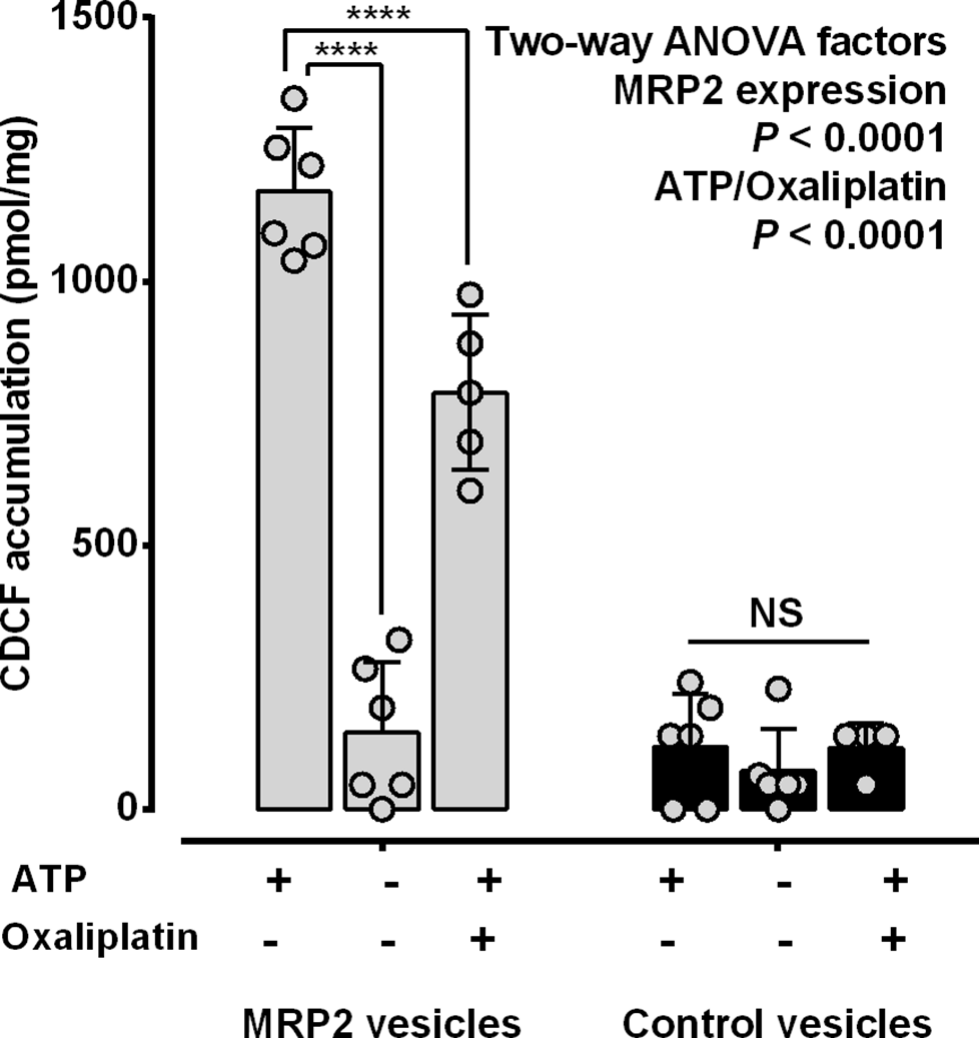
ATP-dependent accumulation of CDCF in MRP2-expressing membrane vesicles: inhibition by oxaliplatin. MRP2-expressing and control membrane vesicles were incubated with CDCF (5 μM) for 5 min with or without ATP (4 mM) and oxaliplatin (400 μM), before measurement of CDCF accumulation by fluorescence. The *P* values shown as numbers are from two-way ANOVA and those shown as *** (*P*< 0.001) and N.S. (*P*> 0.05) are from Tukey’s multiple comparison post-tests following two-way ANOVA for comparisons with the respective membrane vesicles incubated with ATP but no oxaliplatin. Bars represent the means and standard deviations of individual values (open symbols) pooled from two independent experiments. Grey bars, MRP2-expressing membrane vesicles. Black bars, control membrane vesicles.

To validate the MRP2-mediated membrane transport assay, MRP2 expressing and control membrane vesicles were exposed to a model fluorescence probe (CDCF; 5 μM) for 5 min in the presence or absence of ATP (4 mM) with or without oxaliplatin (400 μM), before measurement of CDCF accumulation by fluorescence. Membrane vesicle accumulation of CDCF was significantly increased by 9.4-fold by MRP2 (*P*<0.0001 Two-way ANOVA) and by 7.7-fold by ATP (*P*<0.0001 Two-way ANOVA) as compared to control membrane vesicles and the absence of ATP, respectively (Fig 1). Oxaliplatin significantly inhibited the ATP-dependent accumulation of CDCF in MRP2-expressing membrane vesicles by 25 ± 15% (*P*<0.0001 Tukey’s multiple comparison post-test following two-way ANOVA) but not in control membrane vesicles.

**Fig 2:**
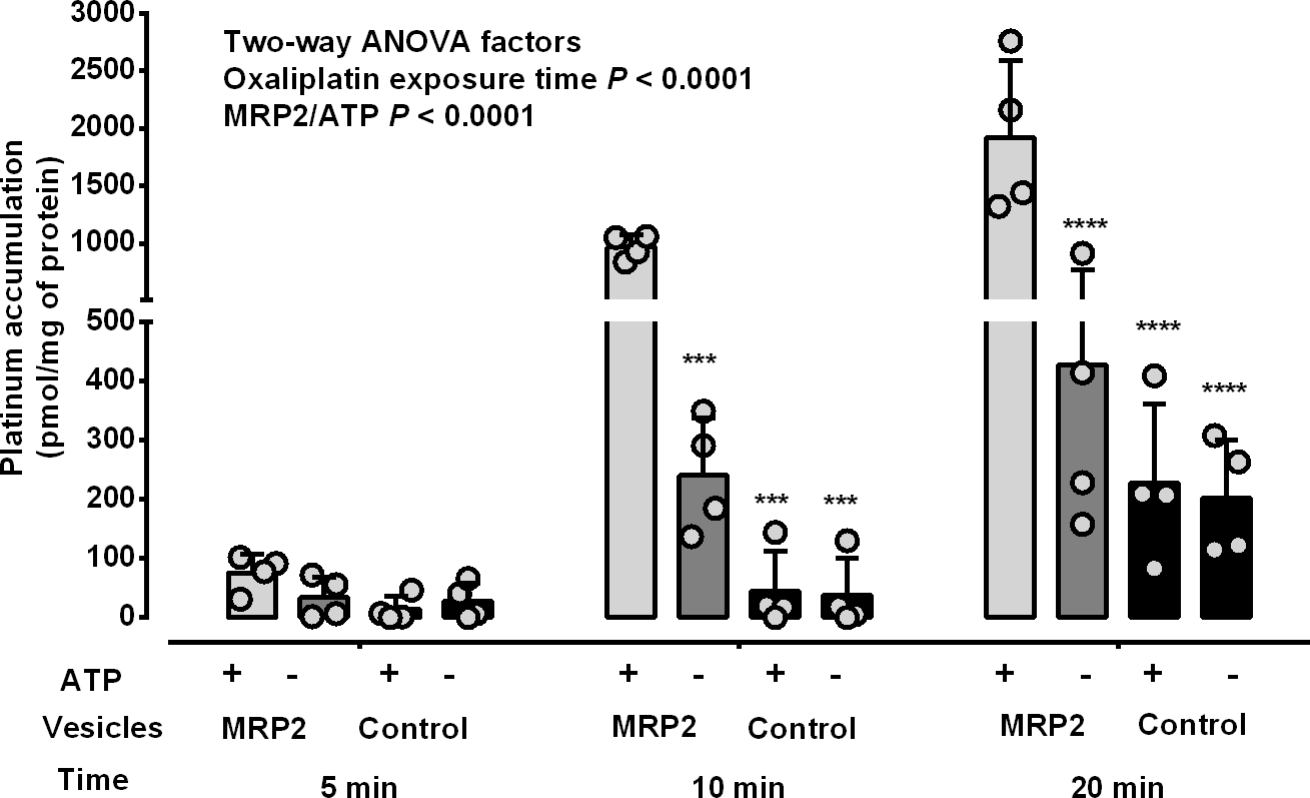
Membrane vesicle accumulation of oxaliplatin-derived platinum: dependence upon MRP2, ATP and oxaliplatin exposure time. MRP2-expressing and control membrane vesicles were incubated with oxaliplatin (100 μM), with or without ATP (4 mM) for 5, 10 and 20 min, followed by measurement of platinum accumulation by ICPMS. The *P* values shown as numbers are from two-way ANOVA and those shown as *** (*P*< 0.001) and **** (*P* < 0.0001) are from Tukey’s multiple comparisons post-tests following two-way ANOVA for comparisons with MRP2-expressing membrane vesicles incubated with ATP at each respective time point. Bars represent means and standard deviations of individual values (open symbols) pooled from two independent experiments. Light grey bars, MRP2-expressing membrane vesicles with ATP. Dark grey bars, MRP2-expressing vesicles without ATP. Black bars, control membrane vesicles.

To investigate the role of MRP2 in the membrane transport of oxaliplatin, MRP2-expressing and control membrane vesicles were incubated with oxaliplatin (100 μM) with or without ATP (4 mM) for 5, 10 or 20 min followed by measurement of platinum accumulation by ICPMS. Membrane vesicle platinum accumulation increased with increasing oxaliplatin exposure time independently of MRP2 expression and ATP (*P*<0.0001 Two-way ANOVA) (Fig 2). Membrane vesicle platinum accumulation was significantly increased by MRP2 expression and ATP as compared to control membrane vesicles and the absence of ATP, respectively (*P*<0.0001 Two-way ANOVA). After 5 min exposure, membrane vesicle platinum accumulation was similar in MRP2-expressing and control membrane vesicles, and in the presence and absence of ATP. However, at 10 and 20 min, membrane vesicle platinum accumulation was increased by MRP2 expression and ATP, by 4- to 19-fold, as compared to control membrane vesicles and the absence of ATP, respectively (*P*<0.001 Tukey’s multiple comparison post-test following two-way ANOVA).

**Fig 3:**
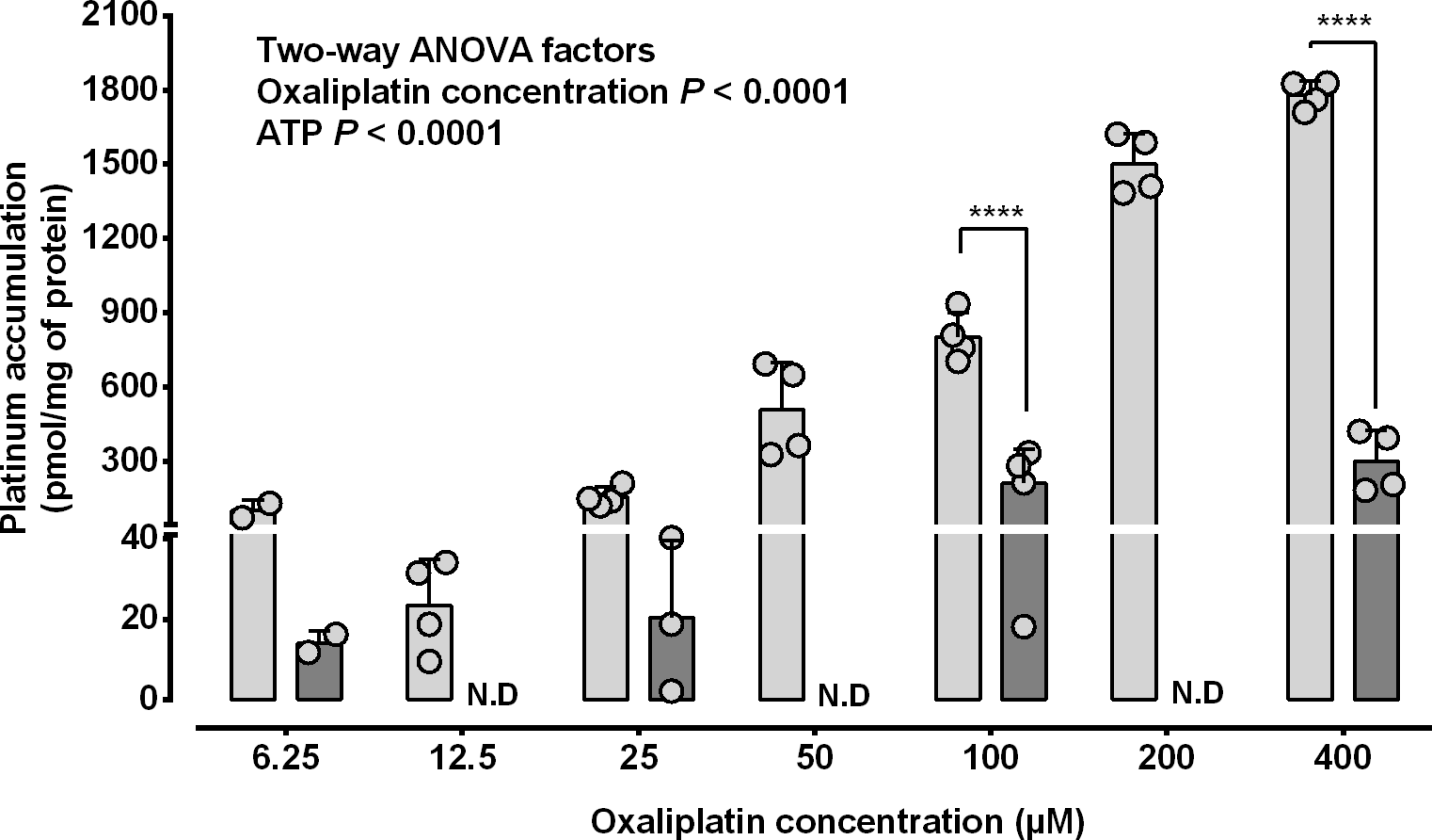
MRP2-mediated membrane vesicle accumulation of oxaliplatin-derived platinum: dependence upon oxaliplatin exposure concentration and ATP. MRP2-expressing membrane vesicles were incubated with oxaliplatin (6.25 to 400 μM) with or without ATP (4 mM) for 10 min before measurement of platinum accumulation by ICPMS. The *P* values shown as numbers are from two-way ANOVA and those shown as **** (*P*< 0.0001) are from Tukey’s multiple comparison post-tests following two-way ANOVA. The bars represent means and standard deviations from individual values (open symbols) pooled from two independent experiments. Light grey bars, with ATP. Dark grey bars, without ATP.

**Fig 4:**
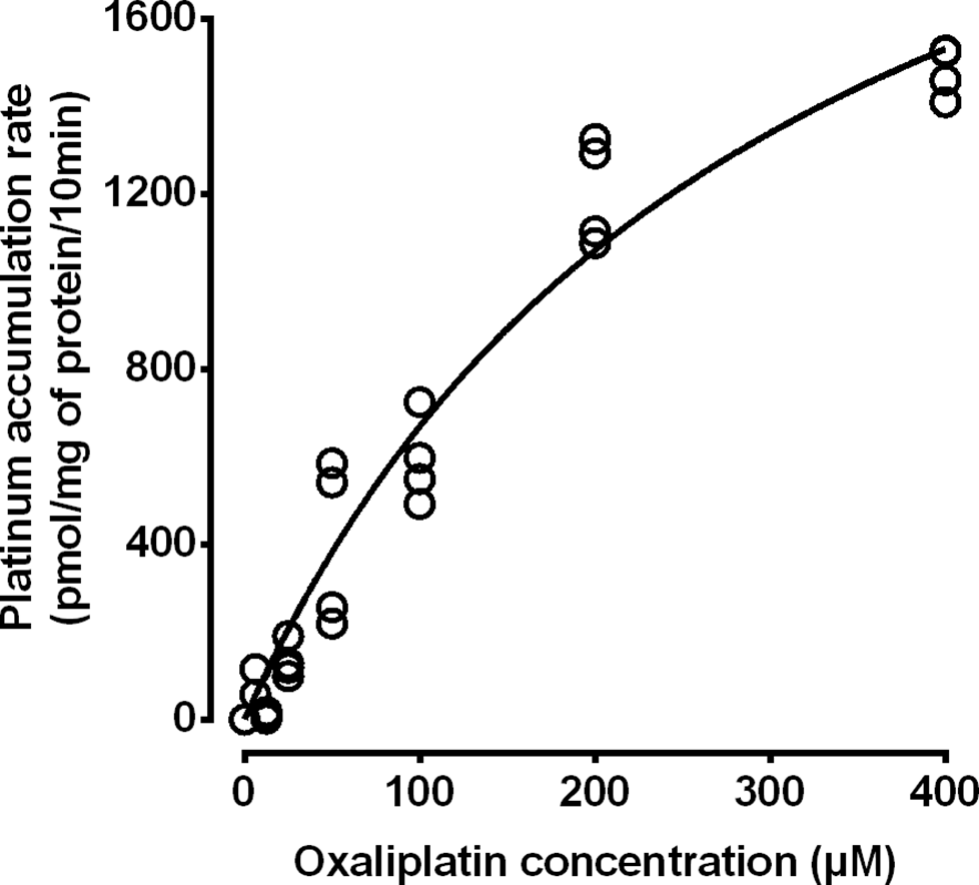
Kinetic analysis of MRP2-mediated active transport of oxaliplatin-derived platinum. Rates of ATP-dependent platinum accumulation in MRP2-expressing membrane vesicles were derived, plotted against oxaliplatin exposure concentration and fitted to a non-linear model. Symbols represent individual values pooled from two independent experiments. The line represents a nonlinear Michaelis-Menten regression fit (r2 = 0.954) with a Vmax of 2680 pmol Pt/mg protein/10 min (95%CI, 2010 to 3360 pmol Pt/mg protein/10 min) and a Km of 301 μM (95% CI, 163 to 438 μM).

The concentration dependence of MRP2-mediated active transport of oxaliplatin-derived platinum was investigated by incubating MRP2-expressing membrane vesicles with oxaliplatin at 6.25 to 400 μM, with or without ATP (4 mM) for 10 min followed by measurement of platinum accumulation by ICPMS. Membrane vesicle platinum accumulation increased with increasing oxaliplatin exposure concentration (*P*<0.0001 Two-way ANOVA) (Fig 3). Membrane vesicle platinum accumulation was increased by ATP by up to 5.9-fold compared to the absence of ATP (*P*<0.0001 Two-way ANOVA). The kinetics of MRP2-mediated active transport of oxaliplatin was investigated by plotting rates of ATP-dependent platinum accumulation in MRP2-expressing membrane vesicles against oxaliplatin exposure concentration and fitting these data to a nonlinear model (Fig 4). The rate of ATP-dependent MRP2-mediated active transport of oxaliplatin-derived platinum increased nonlinearly with oxaliplatin exposure concentration, approaching a plateau value (Vmax) of 2680 pmol Pt/mg protein/10 min (95%CI, 2010 to 3360 pmol Pt/mg protein/10 min), with the half-maximal platinum accumulation rate occurring at an oxaliplatin exposure concentration of 301 μM (95% CI, 163 to 438 μM), in accordance with Michaelis-Menten kinetics (r^2^ = 0.954).

**Fig 5:**
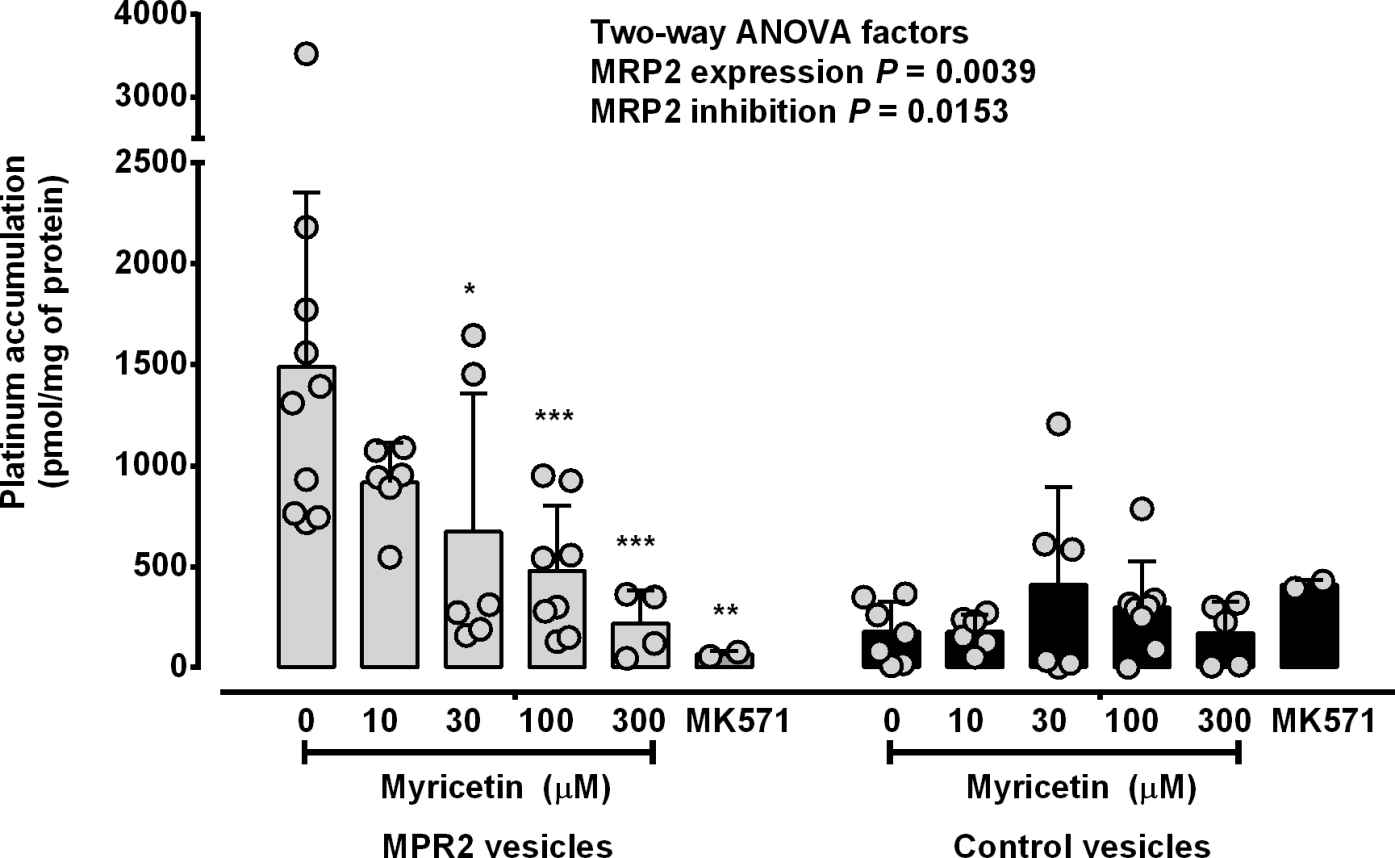
Effects of MRP2 inhibitors on membrane vesicle accumulation of oxaliplatin-derived platinum. MRP2-expressing and control membrane vesicles were incubated with oxaliplatin (100 μM), ATP (4 mM) and MRP2 inhibitors for 10 minutes before measurement of platinum accumulation by ICPMS. The *P* values shown as numbers are from two-way ANOVA and those shown as * (*P* < 0.05), ** (*P* < 0.01) and *** (*P* < 0.001) are from Tukey’s multiple comparison post-tests following two-way ANOVA for comparisons to MRP2-expressing membrane vesicles incubated with oxaliplatin and ATP but without MRP2 inhibitors. Bars represent means and standard deviations of individual values (open symbols) pooled from at least two independent experiments. Light grey bars, MRP2-expressing membrane vesicles. Black bars, control membrane vesicles.

To investigate the effects of MRP2 inhibitors [37–39, 41, 42] on the active transport of oxaliplatin-derived platinum, MRP2-expressing and control membrane vesicles were incubated with oxaliplatin (100 M), ATP (4 mM), myricetin (10 to 300 μM) and MK571 (100 μM) for 10 min before measurement of platinum accumulation by ICPMS. MRP2 inhibitors reduced ATP-stimulated platinum accumulation in MRP2-expressing membrane vesicles in a concentration-dependent manner (*P* = 0.0153 Two-way ANOVA) (Fig 5). Co-treatment with 30, 100 or 300 μM of myricetin or 100 μM of MK571, induced reductions in platinum accumulation in MRP2-expressing membrane vesicles by 58 ± 40%, 78 ± 12%, 85 ± 11% and 96 ± 0.98%, respectively, as compared to no MRP2 inhibitor (*P*< 0.05 Tukey’s multiple comparison post-test following two-way ANOVA). Platinum accumulation in control membrane vesicles was unaltered by myricetin or MK571, but was lower than in MRP2-expressing membrane vesicles (*P* = 0.0039 Two-way ANOVA).

**Fig 6:**
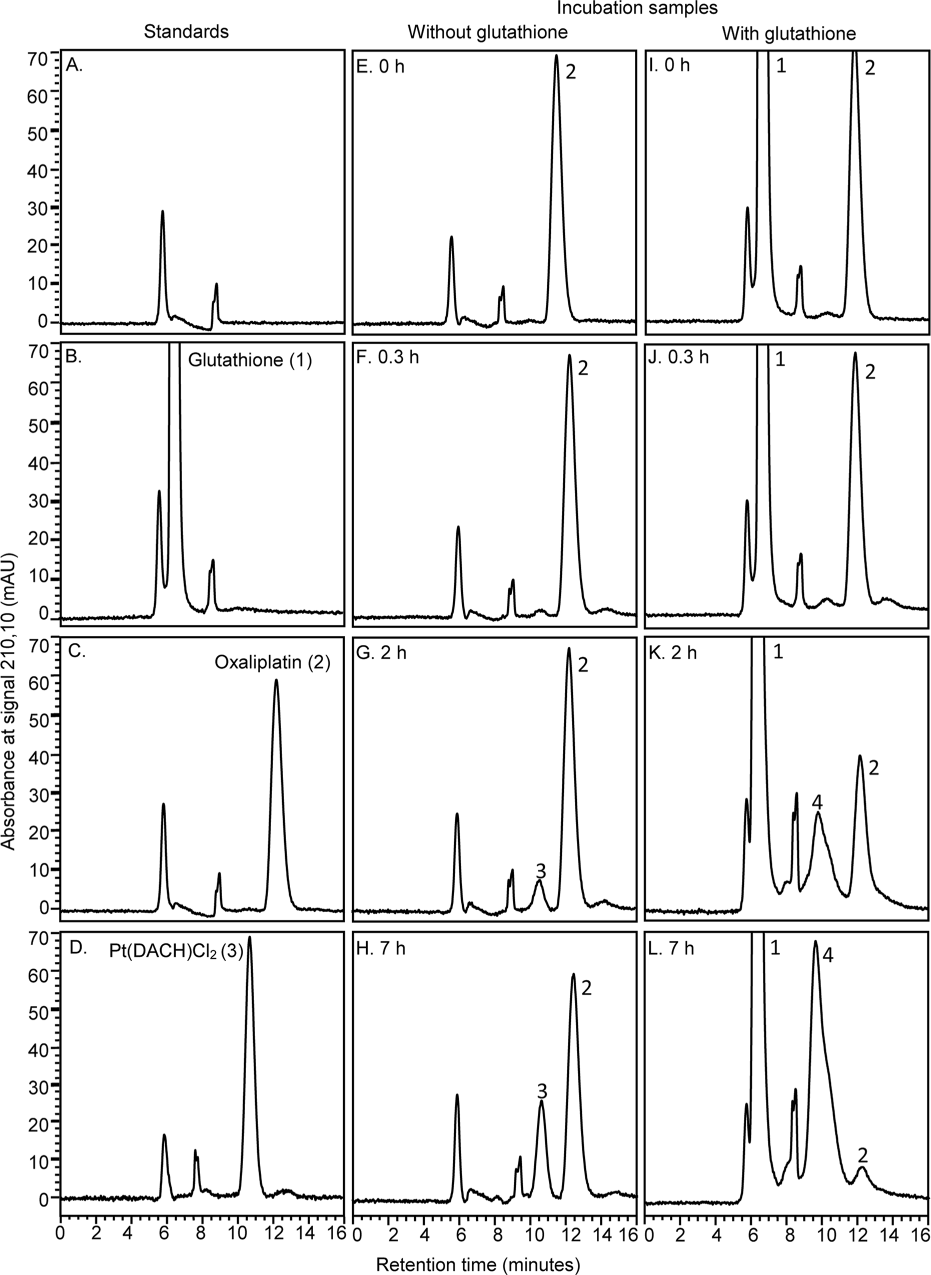
HPLC-UV detection of oxaliplatin, glutathione and degradation products in membrane vesicle incubation buffer. Injection of blank membrane vesicle incubation buffer (A) and authentic standards showed chromatographic separation of glutathione (B, peak 1, retention time 6.5 min), oxaliplatin (C, peak 2, retention time 12.5 min) and Pt(DACH)Cl_2_ (D, peak 3, retention time 10.5 min) with no interference from components of the blank membrane vesicle incubation buffer. Oxaliplatin was incubated (100 μM, pH 7.4, 37˚C) in membrane vesicle incubation buffer with (I-L) or without glutathione (2 mM) (E-H) before analysis of samples by HPLC-UV after 0 hours (E,I), 0.3 hours (F,J), 2 hours (G,K) or 7 hours (H,L) incubation time. After 0.3 hours incubation time (F,J), HPLC-UV chromatograms appeared more-or-less unchanged from the start of the incubation (E,I) and similar with (J) and without glutathione (F). With an increasing incubation time, the oxaliplatin peak areas progressively reduced, and new peaks appeared, corresponding to Pt(DACH)Cl_2_ (G,H: peak 3) in solutions containing no glutathione, and an unknown peak (K,L: peak 4) in solutions containing glutathione. Chromatograms are representative of those from two independent experiments.

**Fig 7:**
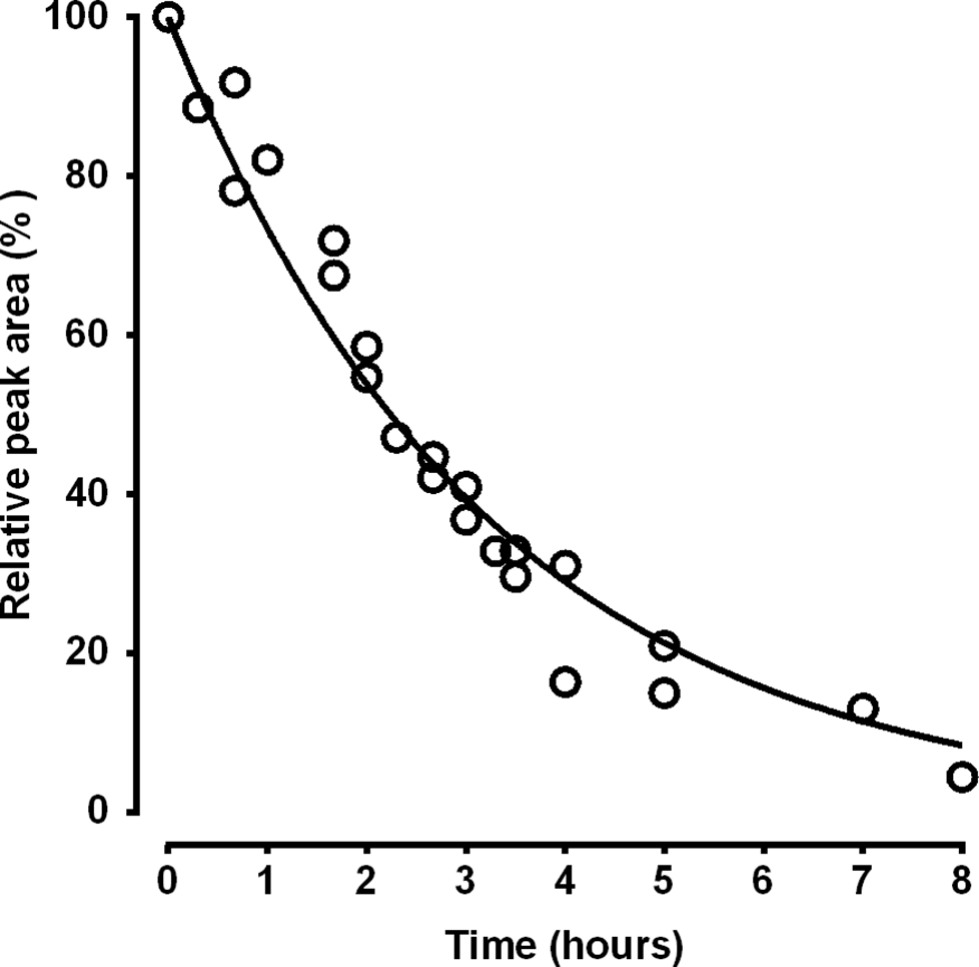
Kinetic analysis of oxaliplatin degradation in membrane vesicle incubation buffer containing glutathione. Open symbols represent individual values of oxaliplatin chromatographic peak areas pooled from two independent experiments. The line represents a nonlinear one phase exponential decay regression fit (r^2^ = 0.964) to the data giving an oxaliplatin degradation half-life of 2.24 hours (95%CI, 2.08 to 2.43 hours).

The possible degradation of oxaliplatin in membrane vesicle incubation buffer during incubations was studied to identify whether oxaliplatin, or perhaps a degradation product, was the likely substrate for this active transport mechanism. HPLC chromatograms of authentic standards of oxaliplatin, glutathione and Pt(DACH)Cl_2_ showed acceptable separation of analytes with retention times of 6.5, 12.5 and 10.5 minutes, respectively, with no interference from any components of blank membrane vesicle incubation buffer, under these HPLC conditions (Fig 6). After incubation of oxaliplatin for 20 min with or without glutathione (pH 7.4, 37˚C), the HPLC-UV chromatograms appeared more-or-less unchanged from the start of the incubation. No new peaks appeared and a very small (10%) reduction in the oxaliplatin peak area was observed at this time point. With an increased incubation time, the oxaliplatin peak areas progressively were reduced and new peaks appeared, corresponding to Pt(DACH)Cl_2_ in the solution without glutathione, and an unknown compound eluting at 9.5 min in the solution with glutathione. Kinetic analysis of the oxaliplatin concentration time-course gave a degradation half-life of 2.24 h (95%CI, 2.08 to 2.43 h) during incubation (pH 7.4, 37˚C) in membrane vesicle incubation buffer containing glutathione (Fig 7). Interpolation of the nonlinear regression fit to the oxaliplatin degradation data indicated that 95% of the added oxaliplatin (95%CI, 94.6 to 95.4%) remained intact after a 10 min incubation, and after 20 min, 90.2% of the added oxaliplatin (95%CI, 89.5 to 90.9%) remained intact, in membrane vesicle incubation buffer containing glutathione.

## Discussion

The findings described in this paper provide direct experimental evidence for a role for MRP2 in mediating the active membrane transport of oxaliplatin-derived platinum. These experiments used a commercially available crude membrane preparation, containing inside-out and right-side-out membrane vesicles expressing MRP2 protein to investigate the active transport of platinum during *in vitro* exposure to oxaliplatin, in the presence or absence of ATP, known substrates and inhibitors of MRP2 and in comparison to control membrane vesicles not expressing MRP2. Like other ABC transporters, MRP2 is thought to transport substrates across cell membranes via changes in protein conformation from a configuration with an internally-accessible high-affinity substrate binding site to one with an externally-accessible lower-affinity substrate binding site, induced by substrate binding, ATP binding and hydrolysis, and dimerization of its nucleotide binding domains [22]. In keeping with this mechanism, the current study showed that the accumulation of platinum was increased with the membrane vesicle expression of MRP2 compared to control membrane vesicles, and by the presence of ATP as compared to no ATP. Furthermore, the rate of active transport of platinum mediated by MRP2 and ATP increased nonlinearly with increasing oxaliplatin exposure concentration approaching a plateau level, consistent with saturation of MRP2-mediated active transport of oxaliplatin-derived platinum. Further evidence included the finding that known MRP2 inhibitors, myricetin and MK571 [37–39, 41, 42], inhibited platinum accumulation, and oxaliplatin inhibited the accumulation of a known MRP2 substrate (CDCF) in MRP2-expressing membrane vesicles, but not in control membrane vesicles. Although IC50 values for myricetin inhibition of MRP2 transport of oxaliplatin-derived platinum were not defined precisely, they appeared to lie between 10 to 100 μM, which was consistent with reported values of myricetin inhibition of MRP2 transport [37–39]. Taken together, these findings provided direct experimental evidence for oxaliplatin interacting with MRP2 protein and being actively transported across cell membrane by a MRP2-mediated and ATP-dependent process.

A finding made in this study, relating to the time course of platinum accumulation in membrane vesicles, was that platinum accumulation increases with increasing oxaliplatin exposure time in both MRP2-expressing and control membrane vesicles and irrespective of the presence or absence of ATP. Mechanisms other than the ATP-dependent active transport of platinum mediated by MRP2 must also be involved in the membrane vesicle accumulation of platinum, which occurred independently of ATP and MRP2 expression. Oxaliplatin is known for irreversibly binding to sulphur residues of proteins with a half-life of approximately one hour [8, 43]. In this way, oxaliplatin may have become non-specifically bound to proteins in MRP2-expressing and control membrane vesicles in an ATP- and MRP2-independent manner. Alternatively, oxaliplatin may have been taken up by membrane vesicles via passive diffusion in a manner also independent of ATP and MRP2. Oxaliplatin protein binding and passive diffusion would also be expected to occur independently of the inside-out or right-side-out orientation of the membrane vesicles.

We observed an apparent time-dependent pattern of ATP-dependent transport of oxaliplatin mediated by MRP2. No differences were found in the accumulation of platinum between MRP2-expressing and control membrane vesicles, in the presence or absence of ATP, after 5 minutes incubation with oxaliplatin. However, after 10 or 20 minutes incubation with oxaliplatin, platinum accumulation was 4- to 19-fold higher in MRP2-expressing membrane vesicles incubated with ATP as compared to control membrane vesicles or the absence of ATP. This finding suggests that the ATP-dependent transport of platinum mediated by MRP2 was delayed in onset until after the first 5 minutes of incubation with oxaliplatin, although further experimentation is required to verify the existence of this possible time-dependent trend. In contrast, the ATP-dependent accumulation of CDCF by MRP2-expressing membrane vesicles was evident after 5 minutes incubation. Previous reports of membrane vesicle studies of other substrates showed almost instantaneous active transport, 30 seconds in the case of [^3^H] leukotrieneC4 transport [42, 44–46] and other radiolabelled substrates [47, 48] in MRP2-expressing membrane vesicles. Mechanisms involved in this apparently delayed onset of ATP-dependent active transport of oxaliplatin-derived platinum are unclear at present and require further study, if this phenomena was confirmed in future experiments. However, time-dependent formation of oxaliplatin degradation products in membrane vesicle incubation buffer that were subsequently transported by MRP2 is a possible explanation for this observation.

Previous studies had shown that oxaliplatin was unstable in aqueous solutions containing chloride or glutathione. In chloride containing solutions, oxaliplatin degrades via oxalate leaving group displacement reactions with chloride ions to form Pt(DACH)Cl_2_ via a monochloro oxalate ring-opened anionic intermediate [Pt(DACH)Cl(oxalate)]^-^ [9]. Reactions between oxaliplatin and glutathione result in the formation of Pt(DACH)glutathione chelates also via oxalate ligand displacement reactions [35]. As the membrane vesicle incubation buffer used in the current study contained both chloride ions (85 mM) and glutathione (2 mM), it could not be assumed that parent oxaliplatin had remained intact or was the form of platinum actively transported by MRP2 under these *in vitro* experimental conditions. To shed light on candidate substrates for MRP2-mediated active transport of oxaliplatin-derived platinum, HPLC-UV studies of oxaliplatin stability and degradation in membrane vesicle incubation buffer were undertaken using conditions designed for separation and detection of intact oxaliplatin and Pt(DACH)Cl_2_ [34]. These studies found that oxaliplatin degraded slowly in membrane vesicle incubation buffer with a degradation half-life of 2.24 hours. Approximately 90% and 95% of the added oxaliplatin had remained intact after 20 and 10 minutes incubation time, respectively. As expected, chromatographic peaks appeared corresponding to Pt(DACH)Cl_2_ and unknown degradation products, which was likely to have been Pt-glutathione chelates, but only after several hours of incubation. These findings point to intact oxaliplatin as a candidate substrate for MRP2 mediated transport, as intact oxaliplatin was the most abundant form of platinum in membrane vesicle incubation buffer under the *in vitro* experimental conditions. Transport of Pt(DACH)Cl_2_ or Pt-glutathione chelates seemed not to account for the MRP2 mediated transport of oxaliplatin demonstrated in this study as neither platinum species appeared to have been formed in the membrane vesicle incubation buffer within the time frame of these studies. However, the monochloro oxalate ring-opened intermediate [Pt(DACH)Cl(oxalate)]^-^, which may not have been detected by the HPLC assay, is another candidate substrate as it is formed early during oxaliplatin degradation reactions with chloride ions [9] and is anionic, which is a property of substrates preferred for MRP2 mediated transport [22]. Further studies of the MRP2-mediated active transport of platinum complexes related to oxaliplatin are required to identify the exact substrates involved in this process.

The pharmacological and clinical significance of these *in vitro* experimental findings is currently unclear and requires further study. MRP2 is expressed on the apical membranes of hepatocytes and proximal renal tubular cells, where it functions in the biliary and renal excretion of endogenous and exogenous compounds [22]. In this way, MRP2 may contribute to the renal and biliary excretion of oxaliplatin, which could be further investigated in MRP2 gene knockout rodent models [49, 50] and clinical studies. Previous studies have linked variations in the DNA base sequence and expression of MRP2 protein with determining the antitumor action and resistance to oxaliplatin in experimental and human tumours [26–30]. Further studies are now required to determine the functional role of MRP2 in limiting tumour cell accumulation of platinum and sensitivity to oxaliplatin, and the therapeutic potential of targeting MRP2 as a strategy for re-sensitizing resistant tumours to chemotherapy [51].

## Conclusions

MRP2 mediates the active transport of platinum derived from oxaliplatin in a manner depending upon ATP. Intact oxaliplatin and its anionic monochloro oxalate ring-opened early degradation product are likely candidate substrates for active transport mediated by MRP2.
